# (*E*)-1-[(2-Chloro-4-nitro­phenyl­imino)­meth­yl]naphthalen-2-ol

**DOI:** 10.1107/S1600536811021374

**Published:** 2011-06-11

**Authors:** Juangang Wang, Jun Zhang, Peipei Yang, Tiedan Chen

**Affiliations:** a College of Chemistry and Materials Science, Huaibei Normal University, Xiangshan, Huaibei 235000, People’s Republic of China

## Abstract

In the title compound, C_17_H_11_ClN_2_O_3_, an intra­molecular O—H⋯N hydrogen bond influences the mol­ecular conformation; the naphthol system and the substituted benzene ring form a dihedral angle of 3.5 (1)°. In the crystal, weak inter­molecular C—H⋯O hydrogen bonds link mol­ecules into chains in the [010] direction The crystal packing exhibits π–π inter­actions between the aromatic rings from the neighbouring mol­ecules, with a centroid–centroid distance of 3.566 (7) Å.

## Related literature

For general background to Schiff bases, see: Caligaris *et al.* (1972 ▶); Salman *et al.* (1990 ▶); Popovic *et al.* (2001 ▶); Garnovskii *et al.* (1993 ▶); Pyrz *et al.* (1985 ▶). For related structures, see: Burgess *et al.* (1999 ▶); Gayathri *et al.* (2007 ▶).
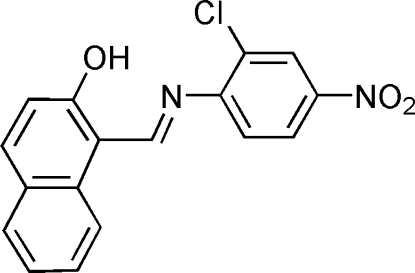


## Experimental

### 

#### Crystal data

C_17_H_11_ClN_2_O_3_
*M* *_r_* = 326.73
Monoclinic, 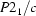

*a* = 7.0530 (8) Å
*b* = 12.8699 (13) Å
*c* = 15.7701 (17) Å
β = 98.180 (1)°
*V* = 1416.9 (3) Å^3^
*Z* = 4
Mo *K*α radiation
μ = 0.29 mm^−1^
*T* = 298 K
0.38 × 0.13 × 0.10 mm

#### Data collection

Bruker SMART APEX CCD area-detector diffractometer
Absorption correction: multi-scan (*SADABS*; Bruker, 2007 ▶) *T*_min_ = 0.899, *T*_max_ = 0.972
7115 measured reflections
2506 independent reflections
826 reflections with *I* > 2σ(*I*)
*R*_int_ = 0.195

#### Refinement

*R*[*F* ^2^ > 2σ(*F* ^2^)] = 0.080
*wR*(*F* ^2^) = 0.185
*S* = 0.92
2506 reflections
208 parameters
H-atom parameters constrained
Δρ_max_ = 0.30 e Å^−3^
Δρ_min_ = −0.33 e Å^−3^

### 

Data collection: *SMART* (Bruker, 2007 ▶); cell refinement: *SAINT* (Bruker, 2007 ▶); data reduction: *SAINT*; program(s) used to solve structure: *SHELXS97* (Sheldrick, 2008 ▶); program(s) used to refine structure: *SHELXL97* (Sheldrick, 2008 ▶); molecular graphics: *SHELXTL* (Sheldrick, 2008 ▶); software used to prepare material for publication: *SHELXTL* and *publCIF* (Westrip, 2010 ▶).

## Supplementary Material

Crystal structure: contains datablock(s) I, global. DOI: 10.1107/S1600536811021374/cv5095sup1.cif

Structure factors: contains datablock(s) I. DOI: 10.1107/S1600536811021374/cv5095Isup2.hkl

Supplementary material file. DOI: 10.1107/S1600536811021374/cv5095Isup3.cml

Additional supplementary materials:  crystallographic information; 3D view; checkCIF report

## Figures and Tables

**Table 1:** Hydrogen-bond geometry (Å, °)

*D*—H⋯*A*	*D*—H	H⋯*A*	*D*⋯*A*	*D*—H⋯*A*
O1—H1⋯N1	0.82	1.81	2.547 (5)	149
C8—H8⋯O3^i^	0.93	2.50	3.392 (5)	160

Symmetry code: (i) 
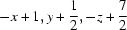
.

**Fig. 1.**
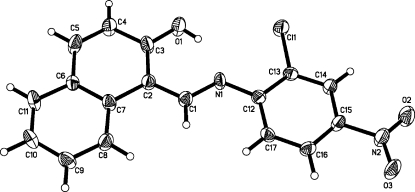
The molecular structure of (I) with displacement ellipsoids shown at 30% probability level.

## supplementary crystallographic information

### Comment

Schiff base ligands play a vital role in coordination chemistry due to their metal binding ability (Garnovskii *et al.*, 1993). In addition, Schiff bases and their metal complexes have wide applications in biological systems (Pyrz *et al.*, 1985). 2-Hydroxy Schiff bases are formed by reactions of salicylaldehyde and 2-hydroxy-1-naphthaldehyde with various amines (Caligaris *et al.*, 1972). In contrast to salicylaldimine derivatives, the Schiff bases of 2-hydroxy-1-naphthaldehyde have been rarely investigated (Salman *et al.*, 1990; Popovic *et al.*, 2001). Herewith we present the title compound (I), derived from 2-hydroxy-1-naphthaldehyde.

In (I) (Fig. 1), the bond lengths and angles are normal and comparable with those observed for unsubstituted analogues (Burgess *et al.*, 1999; Gayathri *et al.*, 2007). Due to intramolecular O—H···N hydrogen bond (Table 1), the C—N=C—C torsion angle (between the phenol and benzene rings) is close to 180° with the value of 178.4 (7)°. In the crystal structure, weak intermolecular C—H···O hydrogen bonds (Table 1) link the molecules into chains in [010]. The crystal packing exhibits π-π interactions between the aromatic rings from the neighbouring molecules with centroid-centroid distance of 3.566 (7) Å.

### Experimental

20 ml of methanol, 2-hydroxy-1-naphthaldehyde (0.172 g, 1 mmol), 2-chloro-4-nitrobenzenamine (0.172 g, 1 mmol) and four drops of acetic acid were added to a 50 ml round bottom flask with a magnetic stir bar. The solution was refluxed for 1.5 h until it was a bright orange color. The solution was then gravity filtered hot and allowed to slowly cool, yielding 0.268 g (78% yield) of bright orange-yellow needle-like crystals.

### Refinement

H atoms were placed in idealized positions (C—H 0.95 - 0.98 Å, O—H 0.82 Å), and thereafter treated as riding , with *U*_iso_ (H) = 1.2-1.5 *U*_eq_ of the parent atom. In view of poor quality of the single-crystal sample selected for data collection (though it was the best one), the relatively high values of *R_int_* and *R*(*F*^2^) were obtained.

### Crystal data

**Table:**

C_17_H_11_ClN_2_O_3_	*F*(000) = 672
*M**_r_* = 326.73	*D*_x_ = 1.532 Mg m^−^^3^
Monoclinic, *P*2_1_/*c*	Mo *K*α radiation, λ = 0.71073 Å
*a* = 7.0530 (8) Å	Cell parameters from 381 reflections
*b* = 12.8699 (13) Å	θ = 2.6–17.6°
*c* = 15.7701 (17) Å	µ = 0.29 mm^−^^1^
β = 98.180 (1)°	*T* = 298 K
*V* = 1416.9 (3) Å^3^	Needle-like, orange
*Z* = 4	0.38 × 0.13 × 0.10 mm

### Data collection

**Table:**

Bruker SMART APEX CCD area-detector diffractometer	2506 independent reflections
Radiation source: fine-focus sealed tube	826 reflections with *I* > 2σ(*I*)
graphite	*R*_int_ = 0.195
φ and ω scans	θ_max_ = 25.0°, θ_min_ = 2.6°
Absorption correction: multi-scan (*SADABS*; Bruker, 2007)	*h* = −8→8
*T*_min_ = 0.899, *T*_max_ = 0.972	*k* = −15→13
7115 measured reflections	*l* = −18→16

### Refinement

**Table:**

Refinement on *F*^2^	Primary atom site location: structure-invariant direct methods
Least-squares matrix: full	Secondary atom site location: difference Fourier map
*R*[*F*^2^ > 2σ(*F*^2^)] = 0.080	Hydrogen site location: inferred from neighbouring sites
*wR*(*F*^2^) = 0.185	H-atom parameters constrained
*S* = 0.92	*w* = 1/[σ^2^(*F*_o_^2^) + (0.0279*P*)^2^] where *P* = (*F*_o_^2^ + 2*F*_c_^2^)/3
2506 reflections	(Δ/σ)_max_ < 0.001
208 parameters	Δρ_max_ = 0.30 e Å^−^^3^
0 restraints	Δρ_min_ = −0.33 e Å^−^^3^

### Special details

**Table:**

Geometry. All e.s.d.'s (except the e.s.d. in the dihedral angle between two l.s. planes) are estimated using the full covariance matrix. The cell e.s.d.'s are taken into account individually in the estimation of e.s.d.'s in distances, angles and torsion angles; correlations between e.s.d.'s in cell parameters are only used when they are defined by crystal symmetry. An approximate (isotropic) treatment of cell e.s.d.'s is used for estimating e.s.d.'s involving l.s. planes.
Refinement. Refinement of *F*^2^ against ALL reflections. The weighted *R*-factor *wR* and goodness of fit *S* are based on *F*^2^, conventional *R*-factors *R* are based on *F*, with *F* set to zero for negative *F*^2^. The threshold expression of *F*^2^ > σ(*F*^2^) is used only for calculating *R*-factors(gt) *etc*. and is not relevant to the choice of reflections for refinement. *R*-factors based on *F*^2^ are statistically about twice as large as those based on *F*, and *R*- factors based on ALL data will be even larger.

### Fractional atomic coordinates and isotropic or equivalent isotropic displacement parameters (Å^2^)

**Table:**

	*x*	*y*	*z*	*U*_iso_*/*U*_eq_	
Cl1	0.2762 (3)	0.18899 (12)	1.41156 (10)	0.0618 (7)	
N1	0.2572 (8)	0.4009 (4)	1.4787 (3)	0.0468 (16)	
N2	0.5023 (10)	0.0960 (4)	1.7252 (4)	0.065 (2)	
O1	0.1367 (7)	0.4326 (3)	1.3208 (3)	0.0730 (18)	
H1	0.1721	0.3999	1.3648	0.110*	
O2	0.4914 (9)	0.0043 (4)	1.7011 (3)	0.0845 (19)	
O3	0.5720 (9)	0.1209 (3)	1.7972 (3)	0.086 (2)	
C1	0.2399 (9)	0.5024 (5)	1.4905 (4)	0.0436 (19)	
H1A	0.2700	0.5291	1.5456	0.052*	
C2	0.1788 (9)	0.5698 (4)	1.4240 (4)	0.0441 (19)	
C3	0.1298 (11)	0.5312 (5)	1.3387 (4)	0.058 (2)	
C4	0.0772 (10)	0.6039 (5)	1.2683 (4)	0.060 (2)	
H4	0.0473	0.5789	1.2127	0.072*	
C5	0.0714 (10)	0.7060 (5)	1.2830 (4)	0.060 (2)	
H5	0.0401	0.7506	1.2366	0.072*	
C6	0.1122 (9)	0.7501 (4)	1.3681 (4)	0.0421 (18)	
C7	0.1664 (9)	0.6842 (4)	1.4387 (4)	0.0441 (19)	
C8	0.2087 (9)	0.7311 (5)	1.5193 (4)	0.052 (2)	
H8	0.2425	0.6894	1.5672	0.062*	
C9	0.2017 (10)	0.8362 (5)	1.5295 (5)	0.054 (2)	
H9	0.2331	0.8656	1.5836	0.065*	
C10	0.1475 (10)	0.8988 (5)	1.4589 (5)	0.056 (2)	
H10	0.1407	0.9704	1.4661	0.068*	
C11	0.1037 (10)	0.8572 (4)	1.3787 (5)	0.054 (2)	
H11	0.0687	0.9002	1.3316	0.065*	
C12	0.3140 (9)	0.3279 (4)	1.5437 (4)	0.0400 (18)	
C13	0.3278 (9)	0.2238 (5)	1.5197 (4)	0.0413 (18)	
C14	0.3845 (10)	0.1468 (4)	1.5788 (4)	0.046 (2)	
H14	0.3892	0.0775	1.5624	0.055*	
C15	0.4332 (9)	0.1761 (5)	1.6617 (4)	0.0444 (18)	
C16	0.4224 (9)	0.2773 (5)	1.6893 (4)	0.047 (2)	
H16	0.4532	0.2941	1.7470	0.057*	
C17	0.3651 (9)	0.3523 (4)	1.6296 (4)	0.0426 (19)	
H17	0.3604	0.4212	1.6470	0.051*	

### Atomic displacement parameters (Å^2^)

**Table:**

	*U*^11^	*U*^22^	*U*^33^	*U*^12^	*U*^13^	*U*^23^
Cl1	0.0977 (16)	0.0460 (10)	0.0362 (10)	−0.0018 (11)	−0.0089 (10)	−0.0058 (8)
N1	0.064 (4)	0.024 (3)	0.048 (4)	0.000 (3)	−0.005 (3)	0.003 (3)
N2	0.103 (6)	0.042 (4)	0.043 (4)	0.001 (4)	−0.013 (4)	0.005 (3)
O1	0.134 (5)	0.037 (3)	0.039 (3)	−0.007 (3)	−0.019 (3)	−0.001 (2)
O2	0.144 (5)	0.043 (3)	0.060 (4)	0.006 (3)	−0.007 (4)	0.004 (3)
O3	0.148 (6)	0.055 (3)	0.040 (3)	0.009 (3)	−0.036 (4)	0.001 (2)
C1	0.051 (5)	0.036 (4)	0.042 (4)	−0.006 (3)	0.002 (4)	0.002 (3)
C2	0.053 (5)	0.037 (4)	0.040 (4)	−0.001 (3)	−0.002 (4)	0.000 (3)
C3	0.084 (6)	0.033 (4)	0.051 (5)	0.007 (4)	−0.008 (5)	−0.003 (3)
C4	0.094 (7)	0.045 (4)	0.032 (4)	0.000 (4)	−0.025 (4)	−0.002 (3)
C5	0.077 (6)	0.048 (5)	0.049 (5)	−0.005 (4)	−0.013 (4)	0.016 (4)
C6	0.050 (5)	0.035 (4)	0.041 (4)	0.005 (3)	0.004 (4)	0.003 (3)
C7	0.051 (5)	0.032 (4)	0.045 (4)	−0.003 (3)	−0.007 (4)	−0.004 (3)
C8	0.062 (5)	0.047 (4)	0.043 (5)	−0.002 (4)	−0.003 (4)	0.008 (3)
C9	0.060 (6)	0.045 (5)	0.053 (5)	0.001 (4)	−0.003 (4)	−0.015 (4)
C10	0.067 (6)	0.030 (4)	0.072 (6)	0.003 (4)	0.008 (5)	−0.010 (4)
C11	0.059 (5)	0.028 (4)	0.070 (6)	0.005 (3)	−0.009 (5)	0.001 (4)
C12	0.046 (5)	0.036 (4)	0.038 (4)	−0.003 (3)	0.006 (4)	−0.003 (3)
C13	0.051 (5)	0.037 (4)	0.034 (4)	−0.003 (3)	0.002 (4)	−0.008 (3)
C14	0.070 (6)	0.029 (4)	0.035 (4)	−0.007 (3)	−0.007 (4)	−0.004 (3)
C15	0.055 (5)	0.042 (4)	0.033 (4)	−0.006 (4)	−0.004 (4)	0.008 (3)
C16	0.062 (5)	0.045 (4)	0.031 (4)	−0.004 (4)	−0.002 (4)	−0.003 (3)
C17	0.053 (5)	0.032 (4)	0.041 (4)	0.001 (3)	−0.001 (4)	−0.008 (3)

### Geometric parameters (Å, °)

**Table:**

Cl1—C13	1.751 (6)	C6—C7	1.408 (8)
N1—C1	1.328 (7)	C7—C8	1.400 (8)
N1—C12	1.406 (7)	C8—C9	1.364 (8)
N2—O3	1.215 (6)	C8—H8	0.9300
N2—O2	1.239 (6)	C9—C10	1.383 (9)
N2—C15	1.471 (7)	C9—H9	0.9300
O1—C3	1.303 (7)	C10—C11	1.368 (9)
O1—H1	0.8200	C10—H10	0.9300
C1—C2	1.383 (8)	C11—H11	0.9300
C1—H1A	0.9300	C12—C17	1.387 (8)
C2—C3	1.429 (8)	C12—C13	1.399 (7)
C2—C7	1.495 (8)	C13—C14	1.380 (7)
C3—C4	1.458 (8)	C14—C15	1.357 (8)
C4—C5	1.335 (8)	C14—H14	0.9300
C4—H4	0.9300	C15—C16	1.379 (8)
C5—C6	1.449 (8)	C16—C17	1.370 (7)
C5—H5	0.9300	C16—H16	0.9300
C6—C11	1.391 (7)	C17—H17	0.9300
			
C1—N1—C12	125.2 (5)	C7—C8—H8	119.0
O3—N2—O2	122.5 (5)	C8—C9—C10	119.5 (7)
O3—N2—C15	120.1 (6)	C8—C9—H9	120.2
O2—N2—C15	117.3 (6)	C10—C9—H9	120.2
C3—O1—H1	109.5	C11—C10—C9	121.1 (6)
N1—C1—C2	122.4 (6)	C11—C10—H10	119.4
N1—C1—H1A	118.8	C9—C10—H10	119.4
C2—C1—H1A	118.8	C10—C11—C6	119.3 (7)
C1—C2—C3	120.3 (6)	C10—C11—H11	120.3
C1—C2—C7	121.2 (6)	C6—C11—H11	120.3
C3—C2—C7	118.5 (5)	C17—C12—C13	117.5 (5)
O1—C3—C2	122.1 (6)	C17—C12—N1	124.7 (5)
O1—C3—C4	118.3 (6)	C13—C12—N1	117.8 (6)
C2—C3—C4	119.6 (6)	C14—C13—C12	122.0 (6)
C5—C4—C3	120.7 (6)	C14—C13—Cl1	118.2 (5)
C5—C4—H4	119.6	C12—C13—Cl1	119.7 (5)
C3—C4—H4	119.6	C15—C14—C13	117.3 (6)
C4—C5—C6	122.6 (6)	C15—C14—H14	121.3
C4—C5—H5	118.7	C13—C14—H14	121.3
C6—C5—H5	118.7	C14—C15—C16	123.3 (6)
C11—C6—C7	120.9 (6)	C14—C15—N2	118.3 (6)
C11—C6—C5	119.5 (6)	C16—C15—N2	118.3 (6)
C7—C6—C5	119.5 (5)	C17—C16—C15	118.2 (6)
C8—C7—C6	117.2 (6)	C17—C16—H16	120.9
C8—C7—C2	123.8 (6)	C15—C16—H16	120.9
C6—C7—C2	119.0 (5)	C16—C17—C12	121.5 (6)
C9—C8—C7	121.9 (6)	C16—C17—H17	119.2
C9—C8—H8	119.0	C12—C17—H17	119.2
			
C12—N1—C1—C2	−178.4 (7)	C8—C9—C10—C11	−1.1 (11)
N1—C1—C2—C3	0.6 (11)	C9—C10—C11—C6	0.6 (12)
N1—C1—C2—C7	−178.1 (6)	C7—C6—C11—C10	−0.5 (11)
C1—C2—C3—O1	1.4 (12)	C5—C6—C11—C10	−178.2 (7)
C7—C2—C3—O1	−179.9 (6)	C1—N1—C12—C17	−1.8 (11)
C1—C2—C3—C4	−176.2 (7)	C1—N1—C12—C13	−179.0 (6)
C7—C2—C3—C4	2.5 (11)	C17—C12—C13—C14	1.9 (10)
O1—C3—C4—C5	−178.7 (7)	N1—C12—C13—C14	179.2 (6)
C2—C3—C4—C5	−1.0 (11)	C17—C12—C13—Cl1	−177.0 (5)
C3—C4—C5—C6	−1.4 (11)	N1—C12—C13—Cl1	0.4 (8)
C4—C5—C6—C11	179.9 (7)	C12—C13—C14—C15	−2.1 (11)
C4—C5—C6—C7	2.2 (11)	Cl1—C13—C14—C15	176.7 (5)
C11—C6—C7—C8	0.8 (10)	C13—C14—C15—C16	2.2 (11)
C5—C6—C7—C8	178.5 (6)	C13—C14—C15—N2	−177.3 (6)
C11—C6—C7—C2	−178.3 (6)	O3—N2—C15—C14	169.8 (7)
C5—C6—C7—C2	−0.6 (10)	O2—N2—C15—C14	−8.1 (10)
C1—C2—C7—C8	−2.0 (10)	O3—N2—C15—C16	−9.8 (11)
C3—C2—C7—C8	179.3 (7)	O2—N2—C15—C16	172.3 (7)
C1—C2—C7—C6	177.0 (7)	C14—C15—C16—C17	−2.0 (11)
C3—C2—C7—C6	−1.7 (10)	N2—C15—C16—C17	177.5 (6)
C6—C7—C8—C9	−1.2 (11)	C15—C16—C17—C12	1.7 (10)
C2—C7—C8—C9	177.7 (6)	C13—C12—C17—C16	−1.6 (10)
C7—C8—C9—C10	1.4 (11)	N1—C12—C17—C16	−178.8 (6)

### Hydrogen-bond geometry (Å, °)

**Table:**

*D*—H···*A*	*D*—H	H···*A*	*D*···*A*	*D*—H···*A*
O1—H1···N1	0.82	1.81	2.547 (5)	149.
C8—H8···O3^i^	0.93	2.50	3.392 (5)	160.

Symmetry codes: (i) −*x*+1, *y*+1/2, −*z*+7/2.

## References

[bb1] Bruker (2007). *SMART*, *SAINT* and *SADABS* Bruker AXS Inc., Madison, Wisconsin, USA.

[bb2] Burgess, J., Fawcett, J., Russell, D. R., Gilani, S. R. & Palma, V. (1999). *Acta Cryst.* C**55**, 1707–1710.

[bb3] Caligaris, M., Nardin, G. & Randaccio, L. (1972). *Coord. Chem. Rev.* **7**, 385–403.

[bb4] Garnovskii, A. D., Nivorozhkin, A. L. & Minkin, V. I. (1993). *Coord. Chem. Rev.* **126**, 1–69.

[bb5] Gayathri, D., Velmurugan, D., Ravikumar, K., Saravanakumar, D. & Kandaswamy, M. (2007). *Acta Cryst.* E**63**, o2324–o2326.

[bb6] Popovic, Z., Roje, V., Pavlovic, G., Matkovic-Calogovic, D. & Giester, G. (2001). *J. Mol. Struct.* **597**, 39–47.

[bb7] Pyrz, J. W., Roe, A. L., Stern, L. J. & Que, L. Jr (1985). *J. Am. Chem. Soc.* **107**, 614–620.

[bb8] Salman, S. R., Shawkat, S. H. & Al-Obaidi, G. M. (1990). *Can. J. Spectrosc.* **35**, 25–27.

[bb9] Sheldrick, G. M. (2008). *Acta Cryst.* A**64**, 112–122. 10.1107/S0108767307043930 18156677

[bb10] Westrip, S. P. (2010). *J. Appl. Cryst.* **43**, 920–925.

